# Dementia after DBS Surgery: A Case Report and Literature Review

**DOI:** 10.4061/2011/679283

**Published:** 2011-11-29

**Authors:** I. Rektorova, Z. Hummelova, M. Balaz

**Affiliations:** ^1^1st Department of Neurology, St. Anne's University Hospital, Medical School of Masaryk University, Pekarska 53, 656 91 Brno, Czech Republic; ^2^Applied Neuroscience Research Group, Central European Institute of Technology, Masaryk University, 602 00 Brno, Czech Republic

## Abstract

We report the case history of a 75-year-old woman with Parkinson's disease who developed severe cognitive problems after deep brain stimulation (DBS) of the bilateral subthalamic nuclei (STN). After a brief cognitive improvement, the patient gradually deteriorated until she developed full-blown dementia. We discuss the case with respect to the cognitive effects of STN DBS and the possible risk factors of dementia after STN DBS surgery.

## 1. Introduction

Parkinson's disease (PD) is a degenerative disorder. Clinical heterogeneity, progressive motor pattern changes, and variations in the course of the disease are well known [1, 2]. The disease process continues throughout life as there is no spontaneous or treatment-induced remission. PD subtypes change with time, and the progression is nonlinear [3]. The point-prevalence of dementia in PD is close to 30%, and the incidence rate is 4- to 6-times greater than that of age-matched controls. The cumulative prevalence of dementia is at least 75% for PD patients who survive for more than 10 years [4–6].

 Although there is no causal treatment for PD, deep brain stimulation (DBS) of the bilateral subthalamic nuclei (STN) has been shown to be surgically safe in well-selected candidates, and subsequent improvements in dopaminergic drug-sensitive symptoms and reductions in drug doses and dyskinesias are well documented. However, the procedure is associated with adverse effects, mainly neurocognitive and neuropsychiatric, and with side effects created by the spread of stimulation to surrounding structures, depending on the precise location of electrodes [7–9]. The morbidity rates associated with invasive surgery can also be significant, including particularly intracranial bleeding [10, 11].

## 2. Case History

A 61-year-old woman was diagnosed with PD in 1991. She had no family history of PD and she had not been chronically treated for any other medical condition. She worked as a high school teacher until the age of 65.

 The first symptoms of PD included hyposmia, fatigue, and tremor and rigidity of the left lower extremity (LLE). She was first put on selegiline. In 1994, she started L-dopa treatment, which had an excellent effect on the motor symptoms of PD. In 1995, while the daily dose of L-dopa was 500 mg, the first choreodystonic peak-dose dyskinesias appeared on the LLE. After that, different therapies were prescribed as add-on treatments to the L-dopa, including dopamine agonists (pergolide, ropinirole), amantadine, and entacapone. These therapies had transient effects in the alleviation of both the motor symptoms of PD and the motor complications. 

 In 2005, the patient was on a stable antiparkinsonian medication therapy consisting of ropinirole 5 mg tid and L-dopa plus entacapone in alternating doses of 100 mg and 50 mg in six 3-hour intervals starting at 7 am (altogether, six doses; total daily L-dopa dose 450 mg). At that time, the patient suffered either from generalised tremor and rigidity accompanied by severe pain of the whole body or from severe choreodystonic involuntary movements. She stopped leaving her house and was unable to take full care of herself. We examined the patient using the Core Assessment Program for Surgical Interventional Therapies in PD (CAPSIT-PD) [12]. At that time, the patient experienced no falls, no postural instability, no freezing of gait, and there was no history of depression, hallucinations, or delusions. According to a detailed neuropsychological examination in October 2005, she was declared cognitively normal in all domains including memory (as measured by the Wechsler Memory Scale III), executive functions (as assessed by Mattis Dementia Rating Scale, semantic and phonological verbal fluency, Tower of London Task, Stroop test), visuospatial and visuoconstructive abilities (as assessed by Rey-Osterrieth Complex Figure Test), and speech [13]. The Mattis Dementia Rating Scale score was 144 points, and her IQ as measured by the Wechsler Adult Intelligence Scale-revised (WAIS-R) was 129 [13]. No symptoms of behavioural or affective disorders were present. The brain MRI was normal for age. Except for PD, the patient was otherwise healthy. She was very motivated for PD surgery. The L-dopa test was clearly positive: the Unified Parkinson's Rating Scale (UPDRS, [14]) III subscore dropped from 44 to 22 points. Despite her high age (75 years), she was considered a good candidate for the procedure.

Bilateral STN electrodes were implanted in December 2005. The stereotactic procedure was performed using the Leibinger open frame with the Praezis Plus software and the Talairach diagram. We used the standard tungsten microelectrode 291A (Medtronic, Inc., Denmark) with an impedance of 0.5–1.5 MΩ for the intraoperative microrecording and microstimulation. Once the target coordinates were determined, a permanent quadripolar DBS electrode (Medtronic, model 3389 with 0.5 mm intercontact distance and 1.5 mm electrode contact width) was implanted. The electrode position was verified by the intraoperative use of fluoroscopy to compare the position of the trajectories of the microrecording electrodes with the definitive trajectory of the quadripolar macroelectrode [15]. The final coordinates of electrode tips within the STN (*x*, *y*, *z*) were 11.5 mm anteriorly, 3 mm posteriorly, 5 mm caudally from AC-PC midpoint. The position of the electrode was confirmed by postoperative CT scan and X-ray with a stereotactic frame still mounted.

 No complications were observed at the time of surgery; the patient remained conscious, alert, and cooperative during all stages of the procedure. Nevertheless, on the day after the electrode implantation, a transient somnolence, disorientation in time and space, and retrograde amnesia occurred. This acute confusion regressed within 4 days. The electrode cables were internalized, and a neurostimulation device (Kinetra, Medtronic Inc., Minneapolis, USA) was implanted. The patient was released from the hospital. She was rehospitalized one month later in order to start the stimulation.

**Figure 1:**
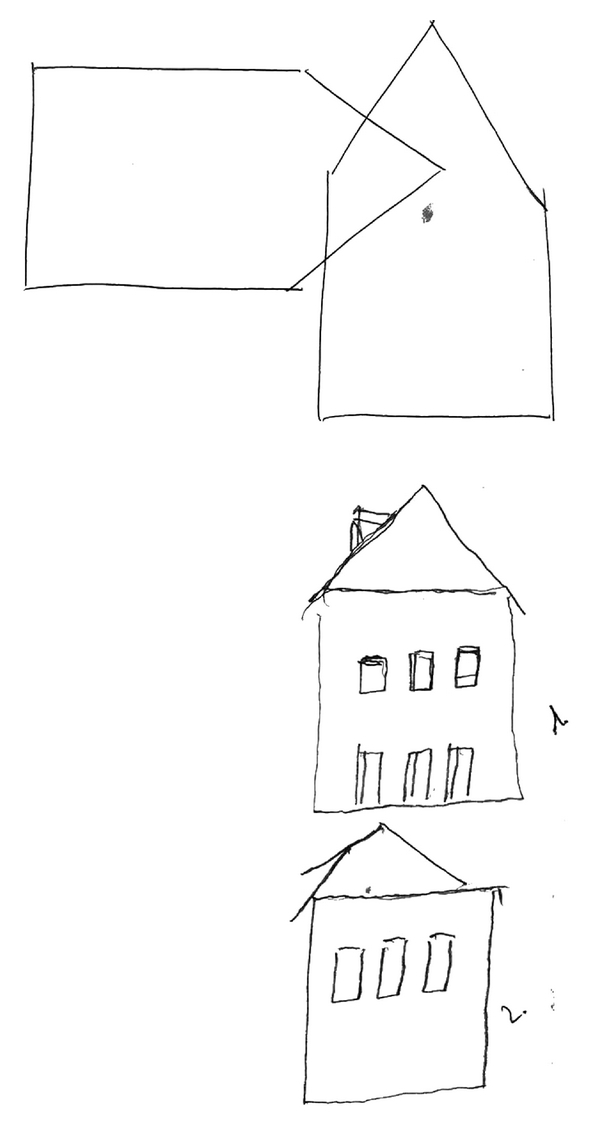
Intersecting pentagons drawing from the Mini-Mental State Examination reflects patient's disinhibition.

**Figure 2:**
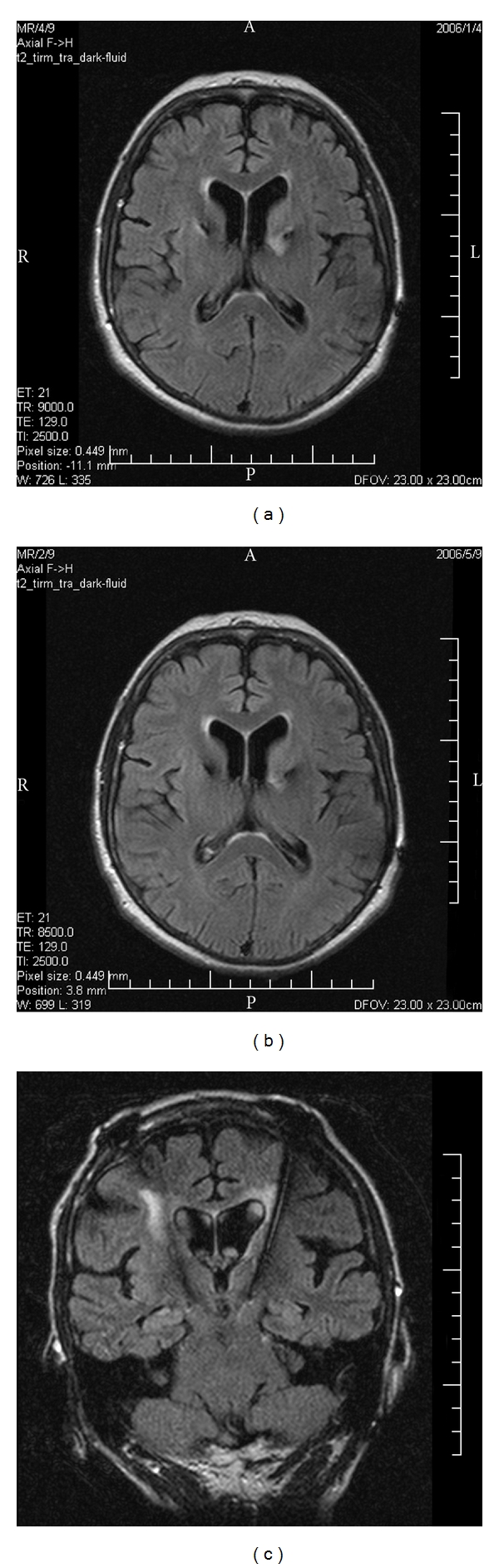
(a) Fluid-attenuated inversion recovery magnetic resonance imaging (FLAIR MRI) in January 2006 shows mild edema and bleeding in the anterior limb of the left internal capsule and left striatum. (b) FLAIR MRI in May 2006 still depicts signal changes along the left electrode trajectory in the internal capsule, medial edge of the lentiform nucleus reaching to the left thalamus. (c) FLAIR MRI in December 2008 demonstrates marked brain atrophy and white matter hyperintensities along the electrode trajectories.

 In January 2006, a neuropsychological examination was performed with the stimulation off while the patient was on stable dopaminergic medication. It revealed a moderately intense organic psychosyndrome and a marked dysexecutive syndrome with a major impact on other cognitive functions and instrumental activities of daily living. It manifested symptomatically by decreased psychomotor speed, flexibility, spontaneity, and concentration, as well as attention deficits and disinhibition (see Figure 1 for the intersecting pentagons drawing from the Mini-Mental State Examination (MMSE, [13])). Mild memory impairment was also identified, affecting primarily recent episodic and semantic memory. Visuospatial deficits, dyscalculia, and deficits in time orientation were also reported, and the patient became negativistic and dysphoric. Her MMSE score was 14 points and the Montgomery-Asberg Depression Rating Scale (MADRS) score was 24 [13]. We performed brain MRI and FLAIR sequences to verify the electrode location and to rule out possible adverse effects of implantation. The electrode location was correct; however, mild oedema and bleeding were found in the anterior limb of the left internal capsule, spreading to the putamen and caudate head (see Figure 2(a)). 

 The stimulation had been started very slowly. By March, the stimulation parameters were set at intensities of 2.0 and 2.7 V, for the left and right sides, respectively, with a 130 Hz frequency, and 90 *μ*sec pulse width. The stimulated contacts were 2 and 6, respectively. The patient's motor symptoms of PD and motor complications improved significantly. The Unified Parkinson's Rating Scale (UPDRS, [15]) III subscore in the off medication state improved from 44 to 21 points. The UPDRS IV subscore decreased from 4 to 0 points. Ropinirole was decreased to 7.5 mg/day, L-dopa was decreased to 300 mg/day (administered in 6 doses), entacapone was 1200 mg/day (6 doses taken together with L-dopa), and citalopram was started (20 mg per day). Cognitive functions had improved slightly from the cognitive outcomes of January 2006. 

 Mild cognitive impairment, with the predominant involvement of the frontal lobes, was reported during neuropsychological testing in June 2006 (see Table 1), affecting verbal fluency, motor sequential tasks, and strategic planning. Mild dyscalculia, recent memory, and visuospatial memory impairments were also identified, but the global cognitive performance was within the normal range; the MMSE score was 27. At that time, the previously described pathology observed on the brain MRI had partially regressed. However, the MRI results in May 2006 still demonstrated hyperintensity changes along the left electrode trajectory in the area of the genu of internal capsule, and the medial edge of the lentiform nucleus reaching to the left thalamus (see Figure 2(b)). On the whole, the clinical status of our patient improved from the motor, behavioural, and cognitive points of view. In June 2006, the stimulation intensity was 3.3 and 3.7 V, for the left and right sides, respectively, while the antiparkinsonian medication remained stable with no changes. We tried to introduce acetylcholinesterase inhibitors (rivastigmine, donepezil) but the patient did not tolerate any of the drugs because of nausea and vomiting.

 During the summer of 2006, the patient's medical condition again started to gradually deteriorate, with episodes of freezing of gait and postural instability, visual hallucinations, and delusions. The behavioural disturbances were worse in the morning. The patient experienced no tremor. Sometimes she had very mild dyskinesias on the left side, but no motor fluctuations were present. She left the house and got lost repeatedly, and she became partially dependent on the caregiver (her husband). Quetiapine was started at that time with a dose titration up to 75 mg per day; this was later exchanged for clozapine in low doses (50 mg per day). All other medications except L-dopa (550 mg/day) and citalopram (20 mg/day) were withdrawn. Cognitive testing in March 2007 confirmed the cognitive decline, with a Mattis Dementia Rating Scale score of 116 points and an IQ score measured at 90. In addition to dysexecutive syndrome, memory functions, and praxis, visuoconstructive, visuospatial, and other cognitive functions were impaired, including writing and picture drawing.

 In 2008, full-blown dementia was reported, which progressed over time. Memantin (20 mg per day) was introduced, but no visible effect on slowing the dementia course was detected. In December 2008, a marked overall brain atrophy was depicted in brain MRI, including both hippocampi. White matter hyperintensities along the electrode trajectories (possible gliosis) were also visible (see Figure 2(c)). In 2009, the MMSE score was 19, and according to the 7-min subtests [13], orientation in time score was 74, enhanced cued memory score was 8, the clock test score was 1, and semantic verbal fluency was 5. In February 2010, the stimulation battery was changed, with the last stimulation parameters being as follows: amplitude 3.6 V and 3.8 V for the left and right sides, respectively, stimulation frequency 130 Hz, pulse width 90 *μ*sec. The MMSE was 14. The patient suffered from hallucinations, delusions, and postural instability with occasional falls. She had severe aphasia and dysarthria with telegraphic slurred speech and moderately severe motor and ideomotor apraxia. She became incontinent, and fully dependent on her husband. She died in April 2010. The probable cause of death was pneumonia. No brain biopsy was performed.

## 3. Discussion

Our patient suffered from intracranial bleeding as a consequence of the STN electrode implantation. Intracerebral hemorrhages (ICHs) have been known to occur as possible adverse effects of DBS surgery in 1 to 4% of cases, according to literature reports of case studies, large studies, and meta-analyses [10, 11, 16–21]. These manifest as transient neurological symptoms, some with complete recovery and others with long-term deficits. In our patient, the specific location of the ICH including the left striatum and thus involving the frontostriatal circuitry could have explained an abrupt cognitive deterioration and marked dysexecutive syndrome in particular [22–24]. The possible risk factors of ICH include particularly arterial hypertension. The impact of the use of microelectrode recordings and of an increased number of microelectrode trajectories have been rather controversial [17–19, 25]. Older age and male sex have also been associated with hemorrhage, according to some studies [19, 26]. Our patient had normal blood pressure; however, her advanced age might have played a role.

 Interestingly, our patient partially recovered, but approximately 8 months after the surgery she again started to deteriorate cognitively until full-blown dementia developed within 2.5 years after the DBS surgery. Although the vast literature on cognitive short-term as well as long-term outcomes after bilateral DBS surgery of the STN varies and remains rather controversial, mild to moderate decreases in verbal fluency have been reported as the most common after-effects of the procedure (e.g., [7–9, 27–32]). The exact mechanisms of possible cognitive effects of STN DBS are not known. The precise location of the active electrode contact and the spatial extent of the effects of stimulation as well as the frequency, voltage, and amplitude of STN stimulation, or patient variables such as degree of dopaminergic denervation could be involved (e.g., [33, 34]).

 Conversely, development of dementia has been generally related to the PD progression itself [30–32]. According to a Sydney study, 48% of PD patients had dementia after 15 years of the disease progression and 83% after 20 years of having PD [5, 35]. Other studies have reported the cumulative prevalence of dementia to be least 75% among PD patients who survive for more than 10 years [36]. The most established risk factors for dementia in PD (PDD) are higher age, severity of motor symptoms, in particular postural and gait disturbances, cognitive impairment at baseline, and visual hallucinations. Other risk factors, such as lower education and socioeconomic status, later disease onset, longer disease duration, positive family history, depression, and REM sleep behavioural disorder have also been reported (e.g., [4–6, 35, 36]). Of all the above-mentioned factors, old age (75 years at the time of DBS and 78 years at the time of dementia diagnosis) and long disease duration (17 years at the time of dementia diagnosis) were the most marked risk factors to take into account in our patient. 

 The time from onset of PD to dementia varies considerably [36, 37]. There is a growing body of evidence derived from clinicopathological studies to suggest that there are different PDD subtypes depending on the age at PD onset and the disease duration. A less malignant type with a long time to dementia onset is characterized by Lewy body distributions consistent with the Braak staging of disease [37, 38], while a more malignant course occurs in people with older age at PD onset and shorter survival shows more brain atrophy and both higher Lewy body and Alzheimer's disease plaque pathology [37]. In our case, the major brain atrophy also seen in the hippocampus, that is, a finding typical of Alzheimer's disease, could have been related to possible coincidence of PD dementia and Alzheimer's pathology and could at least in part explain the malignant course. Unfortunately, the brain biopsy was not performed since the family did not approve it.

 Another interesting issue that has to be taken into consideration relates to possible gliosis along the electrode trajectories. It has been shown by others [39] that DBS electrodes may cause a giant cell reaction or gliosis around them when implanted in the brains of patients with PD. This reaction is present from 3 months to at least 31 months onwards after implantation, and may possibly represent a response to the polyurethane component of the electrodes' surface coat. The accumulation of inflammatory tissue occurs predominantly around the electrode sheath rather than tip, and it is conceivable that on the whole it plays only a small role in maintaining benefit or causing side effects of DBS [39].

 Finally, we indeed cannot exclude a possibility that the ICH as an adverse event of the STN implantation might not only have caused an acute cognitive and behavioural impairment after the procedure but might also have accelerated the development of dementia in our patient, probably as a result of collapsed brain reserve and disturbed compensatory mechanisms caused by the electrode implantation and IHC. Age was probably the major contributor and risk factor for the intracranial bleeding, postoperative confusion, and later dementia development [26, 40, 41]. 

 Despite many unresolved questions, this has taught us not to include PD patients above 70 years of age for the DBS surgery. In addition, the length of PD duration should also be taken into consideration, and the question remains as to what the best time for considering DBS in PD would be. Further research should focus on potential biological markers such as specific brain imaging techniques and cerebrospinal fluid examination that would better predict the disease prognosis and that might help to better select good candidates for DBS surgery in PD patients.

## Figures and Tables

**Table 1:** Neuropsychological test battery results.

Psychological test	Examination date						
	10/2005	1/2006*	3/2006**	6/2006	11/2006	3/2007	7/2008
WAIS-R (IQ)	129	14 MMSE only	102	27 MMSE only	97	90	

Mattis DRS (raw/maximum score)							
Total score	144/144					116/144	84/144
Attention	37/37					35/37	31/37
Initiation	37/37					29/37	14/37
Construction	6/6					5/6	2/6
Conceptualization	39/39					30/39	26/39
Memory	25/25					17/25	11/25

WORD-LIST WMS III (raw/scaled score)							
1st trial	5/12		3/7			2/6	2/6
Immediate recall	29/11		22/7			13/4	15/4
Delayed recall	4/11		4/11			1/8	0/6
Recognition	22/10		23/11			18/7	15/4

Verbal fluency tests (raw score)							
Category (animal)	20		1	14	7	4	6
letter (N, K, P)	45		19	6	10	7	5

Rey-Osterrieth CFT (raw/T score)							
Copy (raw)	36		33		32.5	28	4.5
Immediate recall	21/67		10/43		15/57	12.5/52	1.5/25
Delayed recall	18.5/62		13/49		13.5/54	9/43	failed
Recognition	29/49		20/49		21/56	20/50	14/20

Stroop test (raw/T score)							
Word	98/45			97/45	103/47	93/43	56/24
Color	73/45			61/37	65/40	34/20	31/20
Word/color	49/54			33/38	39/44	25/30	failed
Interference (T score)	57			50	49	55	—

Tower of London (raw/maximum score)	33/36					8/36	failed

MADRS	4	24		9	5	3	8

*1/2006: very poor compliance—only screening; **3/2006: still poor compliance, but much better than during the previous examination in 1/2006; WAIS-R: Wechsler Adult Intelligence Scale—Revised; Mattis DRS: Mattis Dementia Rating Scale; WMS III: Wechsler Memory Scale III; Rey-Osterrieth CFT: Rey-Osterrieth Complex Figure Test; MADRS: Montgomery-Asberg Depression Rating Scale.
